# Case Report: Surgical management of trochlear ridge fracture associated with medial patellar luxation surgery in small-breed dogs

**DOI:** 10.3389/fvets.2026.1732262

**Published:** 2026-04-24

**Authors:** Taro Kimura, Kazuki Sawano, Yuko Kadooka

**Affiliations:** Vet Surg Tokyo, Tokyo, Japan

**Keywords:** medial patellar luxation, screw fixation, small-breed dogs, trochlear recession complications, trochlear ridge fracture

## Abstract

**Introduction:**

In this report of three clinical cases, we describe the diagnosis and surgical management of trochlear ridge fractures occurring during or after medial patellar luxation (MPL) correction in small-breed dogs.

**Case report:**

Three small-breed dogs diagnosed with grade 3 MPL sustained trochlear ridge fractures associated with MPL surgery. In one dog, an iatrogenic fracture occurred intraoperatively during block recession; in contrast, postoperative fractures developed in two dogs approximately 2 months postoperatively after block recession. At follow-up, recurrent patellar luxation was identified, but no trochlear ridge fracture was noted. Each case involved a fracture of the lateral or medial trochlear ridge with preservation of periosteal continuity. Stabilization was performed by placing screws for subfragmentary buttress support beneath the displaced bone fragment to restore ridge integrity. Implant-related complication occurred only in one case that required implant removal due to screw prominence. No dog demonstrated lameness, and resolution of patellar luxation was noted in all dogs with functional recovery maintained for a minimum of 12 months postoperatively.

**Conclusion:**

Screw fixation was used to buttress the displaced trochlear ridge fragment in these cases where periosteal continuity was preserved. Fragment positioning was maintained during the follow-up period in all dogs. This report is descriptive in nature and does not aim to validate biomechanical efficacy or recommend this fixation method over existing techniques.

## 1 Introduction

Trochlear recession is a cartilage-preserving trochleoplasty technique developed to reduce complication rates associated with the surgical correction of medial patellar luxation (MPL), a common orthopedic condition in small-breed dogs (1–3). While complications such as re-luxation, tibial tuberosity displacement, and groove insufficiency are well documented (1, 4), trochlear ridge injury is rarely reported (5), particularly in small breeds. Damage to the medial or lateral trochlear ridge may occur intraoperatively due to technical error or postoperatively due to instability or mechanical stress. Inadequate management of such injuries can compromise joint congruity and long-term function (6). However, evidence directly investigating the configuration or extent of trochlear recession that leads to the development of postoperative trochlear ridge fractures remains limited. Despite its clinical relevance, literature describing the diagnosis and treatment of trochlear ridge fractures in canine MPL surgery is scarce. Furthermore, guidance regarding appropriate fixation methods, especially in small-breed dogs, is lacking. Challenges related to ridge stabilization include limited bone stock, a small contact area, and the risk of implant interference with the joint surface.

This report describes three cases of trochlear ridge injury—one iatrogenic and two postoperative—associated with MPL correction in small-breed dogs and the surgical approach used for management of the fracture. This report is descriptive in nature and does not attempt to establish biomechanical superiority or treatment recommendations.

## 2 Case report

### 2.1 Pilot evaluation

**Figure 1:**
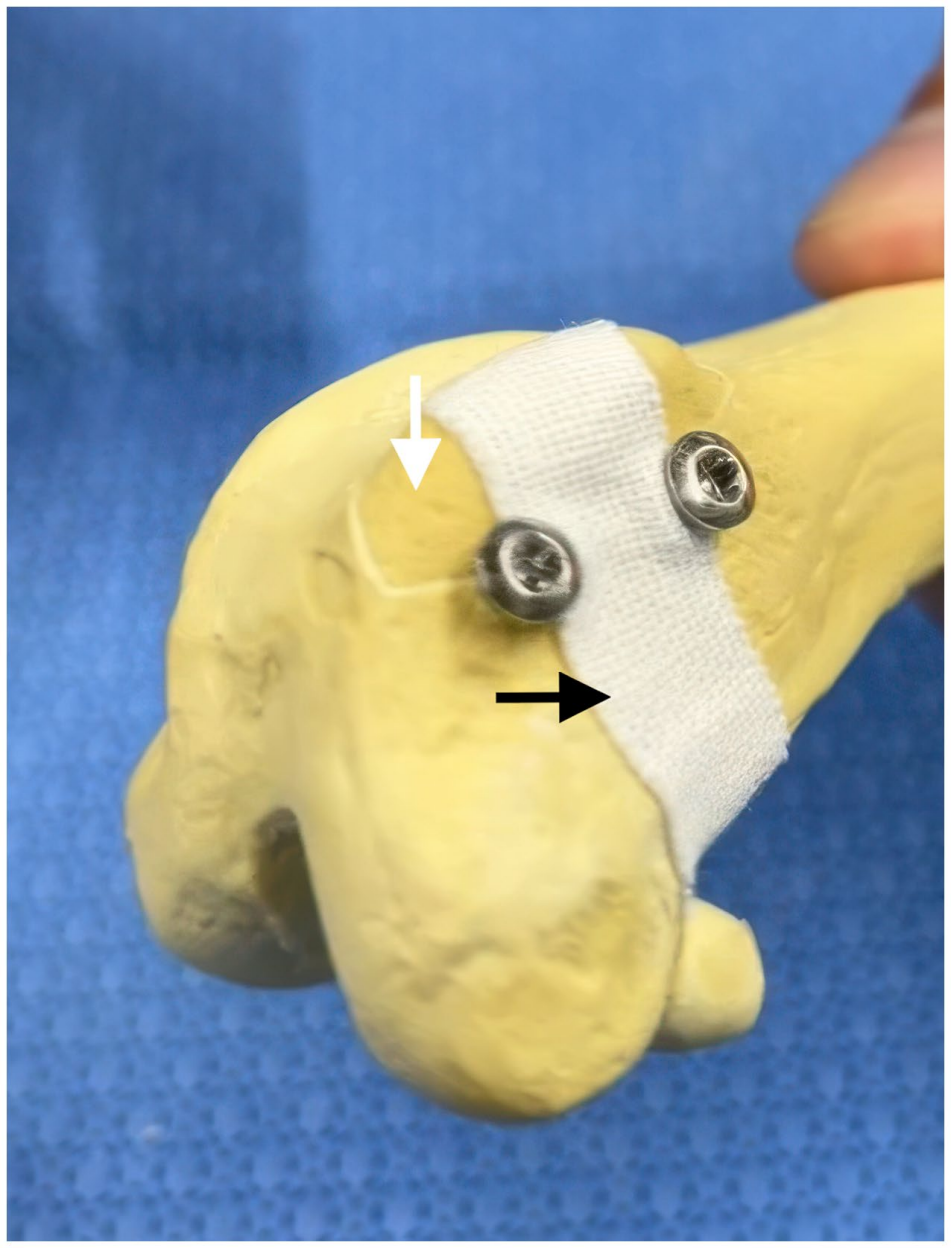
Pilot model illustrating buttress fixation of a trochlear ridge fragment. The white arrow indicates the simulated trochlear ridge fragment, and the black arrow indicates the periarticular soft tissue. Screws are placed beneath the fragment to provide buttress support without penetrating the fragment.

A pilot study using plastic canine femoral models (Figure 1) was performed to assess the feasibility of buttress fixation using two or three subfragmentary screws distributed evenly along its length to provide uniform buttress support placed beneath the trochlear ridge fragment. The pilot evaluation was developed based on prior clinical experience and was not intended as case-specific preoperative planning but rather to establish a fixation concept applicable once a fracture was identified intraoperatively. Based on the pilot model, placement of two or three screws beneath the trochlear ridge fragment was technically feasible in the model. Screws were oriented approximately perpendicular to the local base of the trochlear ridge to maximize subfragmentary support while avoiding intra-articular penetration. No formal mechanical testing was performed. This model was used solely for conceptual planning and did not involve mechanical testing.

### 2.2 Case details and diagnosis

Three small-breed dogs presented with moderate hindlimb lameness and were diagnosed with grade 3 MPL based on orthopedic examination and radiographic evaluation using standard clinical criteria (6). Case 1 was a 5-year-old neutered male Pomeranian (3.6 kg) with a history of lameness since 1 year of age; case 2 involved an 11-month-old spayed female mixed-breed dog (2.5 kg) with a history of lameness since 1 year of age; and case 3 was a 19-month-old intact male Toy Poodle (5.1 kg) who had been lame since she was a year old. The beginning of the lameness was reported by the owners. In each case, MPL correction was elected following failure of conservative management. The initial standard surgical procedures included block recession, which allows approximately 50% of the patella to engage within the groove (2, 7), lateral capsular imbrication (1), lateral anti-rotational suturing using suture anchors and suture materials (3, 4), and tibial tuberosity transposition stabilized with two 0.035- or 0.045-inch Kirschner wires (1, 4). Tension band fixation was not applied due to the small body weight of the dogs. In Cases 1 and 2, revision surgery was performed to address recurrent MPL. Postoperative assessment consisted of orthopedic examination and standard radiography; however, definitive diagnosis of the trochlear ridge fractures was made only at the time of revision surgery. In contrast, in Case 3, the trochlear ridge fracture occurred and was identified intraoperatively.

### 2.3 Ethical considerations

All procedures were conducted in accordance with the accepted veterinary standards. Informed consent was obtained from the owner for all treatments and the publication of the clinical data and images included in this report.

### 2.4 Anesthesia for surgery

The following anesthetic protocol was utilized in these cases: pre-medication was performed using butorphanol, followed by propofol administration for the induction of anesthesia. After endotracheal intubation was achieved, general anesthesia was maintained using isoflurane in 100% oxygen, while a combination of femoral and sciatic nerve block using a compounded lidocaine and bupivacaine solution was administered for analgesia. Cefazolin was administered 30 min prior to surgery and every 90 min intraoperatively as prophylactic antibiotic therapy.

### 2.5 Surgery

Surgical planning was based on the severity of MPL and clinical assessment of limb alignment. Although femoral and tibial deformities were considered, computed tomography and corrective osteotomy were declined by the owners in all cases. Therefore, quantitative morphologic measurements were not obtained. Surgical management was performed without corrective osteotomy. Tibial tuberosity position and alignment were evaluated intraoperatively and addressed by tibial tuberosity transposition as part of the standard MPL correction. For cases 1 and 2, which presented with postoperative medial patellar re-luxation, a standard craniolateral parapatellar approach to the stifle joint was employed during revision surgery. The skin was incised along the craniolateral aspect of the femur, extending from the distal one-third of the femur to just distal to the tibial tuberosity. The biceps femoris fascia was incised, followed by sharp dissection through the lateral joint capsule and the connective tissue attaching the vastus lateralis muscle to the parapatellar fibrocartilage, thereby exposing the stifle joint. This allowed medial luxation of the patella and provided adequate visualization of the trochlear groove. In both cases, fractures were identified at the base of the medial trochlear ridge. Fibrous tissue and inflammatory adhesions were carefully debrided, and the ridge fragment—still attached to the periosteum—was mobilized and anatomically repositioned. In Case 3, which involved an iatrogenic lateral trochlear ridge fracture caused by misdirection of the chisel during block recession, the same surgical approach and reduction technique were employed, and the lateral periosteum remained attached.

**Figure 2:**
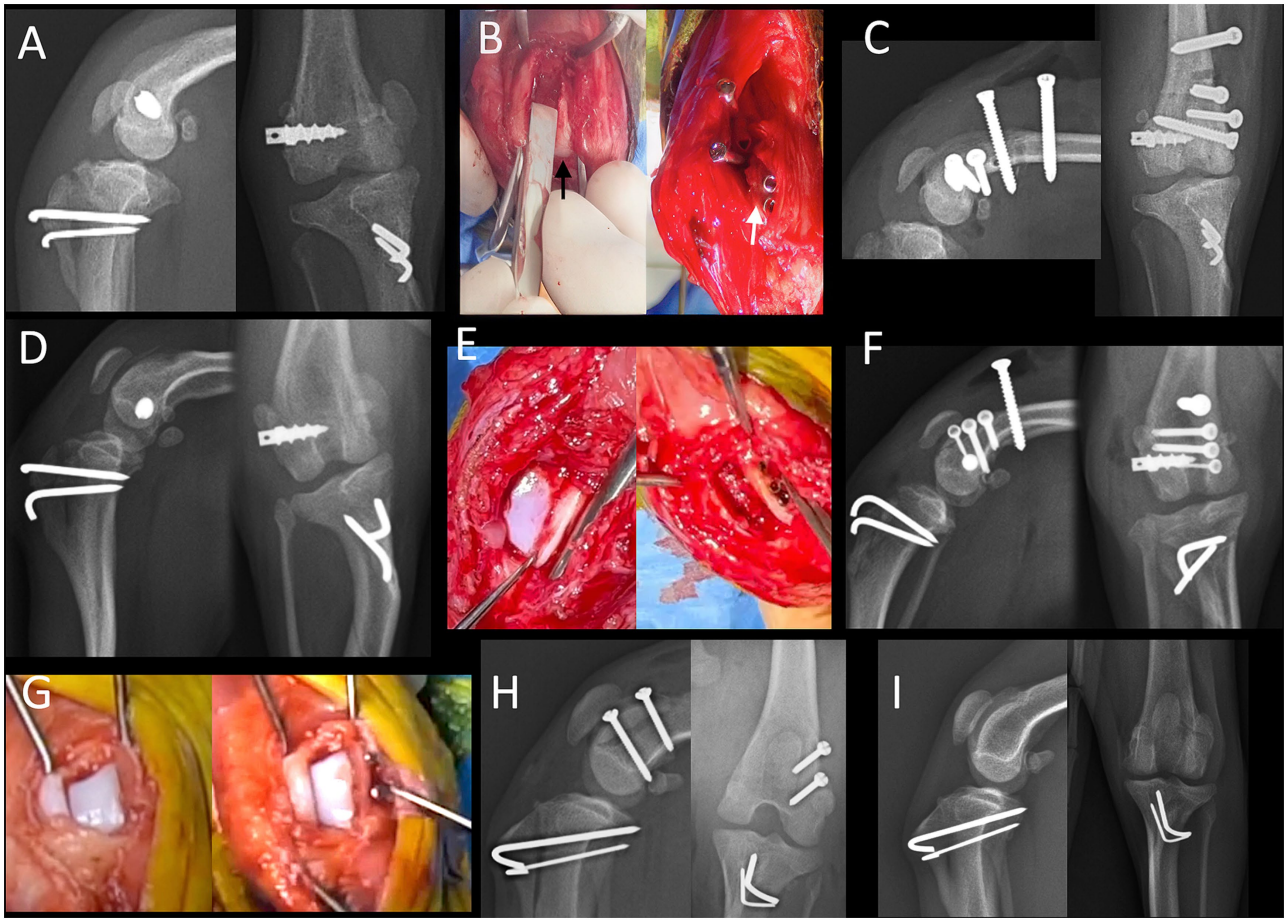
Intraoperative photographs and pre- and postoperative radiographs. **(A,C)** Mediolateral and craniocaudal radiographs from Case 1. **(A)** Preoperative views showing medial patellar re-luxation; no definitive fracture line of the trochlear ridge was clearly identified on standard radiographs. **(C)** Immediate postoperative views demonstrating placement of three subfragmentary buttress screws beneath the trochlear ridge fragment and two additional screws placed to counteract medialising forces. **(B)** Intraoperative photographs from Case 1. Left: fracture line of the medial trochlear ridge prior to fixation (black arrow). Right: reduction of the fragment (white arrow) and subsequent screw fixation. **(D,F)** Mediolateral and craniocaudal radiographs from Case 2. **(D)** Preoperative views demonstrating medial patellar re-luxation; no definitive fracture line of the trochlear ridge was clearly identified on standard radiographs. **(F)** Immediate postoperative views showing reduction of the medial trochlear ridge fragment and placement of subfragmentary buttress screws beneath the fragment and an additional screw placed to counteract medialising forces. **(E)** Intraoperative photographs from Case 2. Left: fractured medial trochlear ridge prior to fixation. Right: reduced and stabilized fragment after screw placement. **(G)** Intraoperative photographs from Case 3. Left: rotated lateral trochlear ridge fragment before reduction. Right: anatomically repositioned and stabilized fragment. **(H)** Immediate postoperative radiographs from Case 3. The screw head appears slightly prominent relative to the cortical surface. **(I)** Postoperative radiograph obtained 377 days after revision surgery (184 days after implant removal) in Case 3.

Surgical management involved placement of two or three subfragmentary screws inserted beneath the fragment to provide buttress support. The number of screws was determined based on fragment length, bone stock, and intraoperative assessment of stability, with two screws used in smaller fragments and three screws used in larger fragments. Screws were oriented approximately perpendicular to the local base of the trochlear ridge to optimize subfragmentary support and avoid intra-articular protrusion, rather than to achieve interfragmentary compression. Three screws were used in Cases 1 and 2, whereas two screws were used in Case 3, reflecting differences in fragment size and available bone stock. Implants used for ridge stabilization included 1.5-mm cortex screws and 2.0-mm locking screws^1^ in Case 1; 1.0-mm and 1.7-mm cortex screws and 1.7-mm locking screws^2^ in Case 2; and a 1.3-mm cortex screw^3^ in Case 3. Screw length was selected to achieve secure purchase while maintaining clearance from the joint space. Kirschner wires^4^ (0.035–0.045 inches) were used temporarily to assist fragment positioning during screw placement and were removed after fixation. No lag-screw technique, interfragmentary compression, or temporary compression maneuver was applied. Intraoperative photographs and pre- and postoperative radiographs from all three cases are presented in Figure 2 to illustrate fracture configuration, fixation method, and postoperative outcome. Clinical details of the three dogs are summarized in Table 1.

**Table 1:** Clinical details of the three dogs included in this study.

Case	Breed	Weight (kg)	Trochlear ridge	Timing of injury	Fixation type	Follow-up (days)	Gait outcome
1	Pomeranian	3.6	Medial	Postoperative (MPL recurrence)	1.5- and 2.0-mm screws	488	Normal
2	Mixed breed	2.5	Medial	Postoperative (MPL recurrence)	1.0-, 1.7-, and 2.0-mm screws	489	Normal
3	Toy Poodle	5.1	Lateral	Intraoperative	1.3-mm screw	377	Normal

MPL, medial patellar luxation.

To reduce the risk of postoperative patellar re-luxation, additional screws were placed to enhance construct stability and counteract traction forces generated by the quadriceps mechanism. Specifically, two 2.0-mm locking screws were used in Case 1 and one 1.7-mm cortex screw was used in Case 2.

### 2.6 Postoperative management

The owners were instructed to confine the dogs to a space in which they could stand and walk on all four limbs without difficulty, with the height limited to prevent them from standing on their hind legs alone. The width of the area was limited to approximately five times the length of the dog’s body, and this restriction was maintained for 4 weeks postoperatively. No other specific rehabilitation protocols were prescribed.

### 2.7 Postoperative assessment

Postoperative evaluations, including physical examinations and radiographic assessments, were conducted at approximately 2, 4, and 8 weeks postoperatively and thereafter at approximately 3-month intervals. Gaits were subjectively assessed by the attending surgeon, and lameness was graded using a scale of 0–5 (8). Radiographs were reviewed to assess implant stability. Osteoarthritic changes were evaluated radiographically based on their presence or absence; fracture healing was not formally assessed radiographically.

### 2.8 Postoperative outcomes

**Figure 3:**
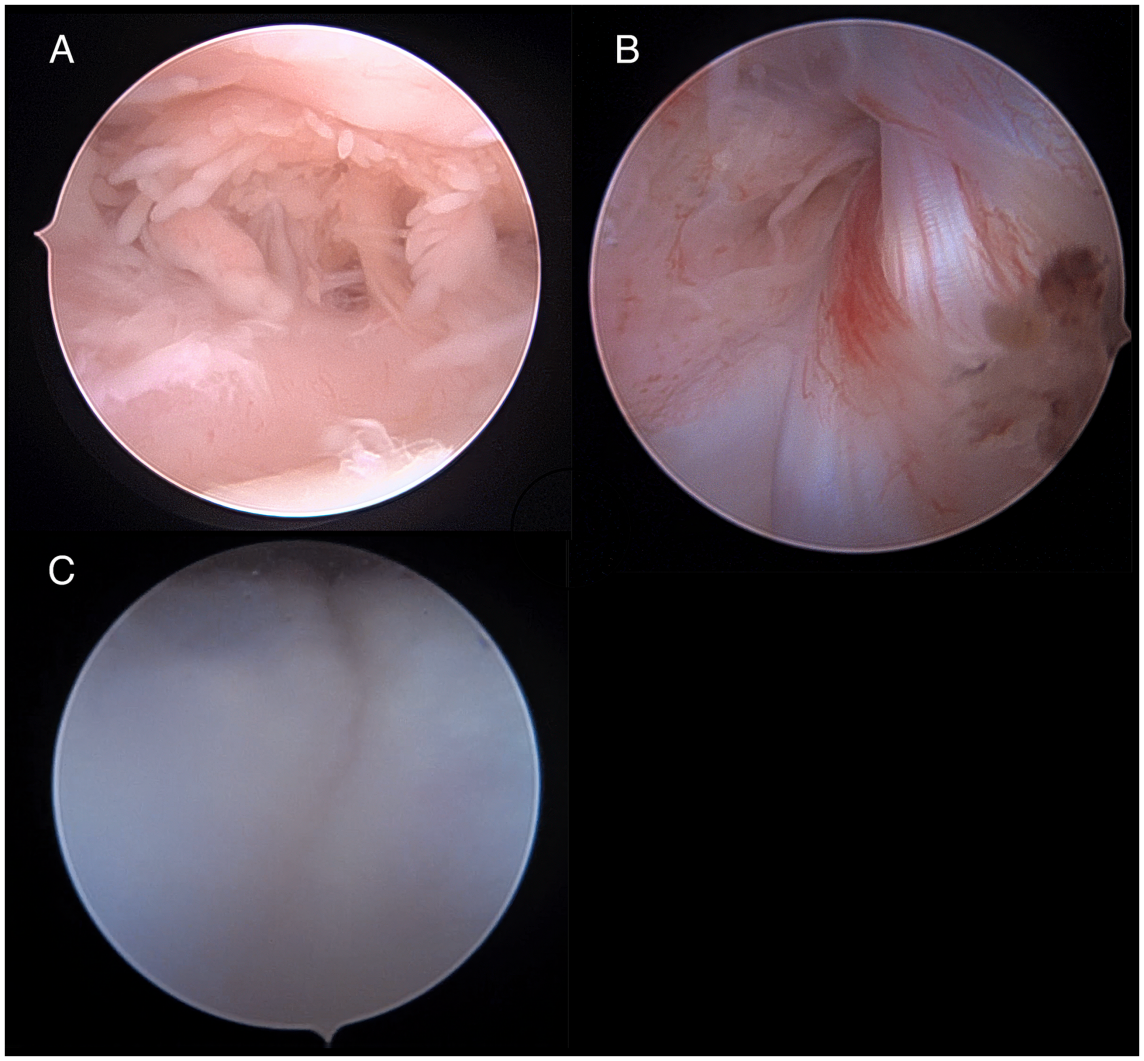
Arthroscopic findings during implant removal in Case 3. **(A)** Severe synovitis is observed at the proximal aspect of the stifle joint capsule, with frond-like proliferation of inflamed synovial tissue. **(B)** Cranial cruciate ligament showing increased vascularization, suggestive of mild inflammatory changes. **(C)** A subtle step-off is noted at the medial aspect of the medial trochlear ridge.

All three dogs underwent surgical management of the trochlear ridge fragment without intraoperative complications. On postoperative craniocaudal radiographs, the anatomical proximal femoral axis was defined by connecting the midpoints of the femoral shaft measured at one-third and one-half of the femoral length from the proximal femur, with each midpoint determined as the midpoint of the shaft width. The trochlear groove axis was defined as a line along the medial trochlear ridge following trochleoplasty. The angle formed between these two axes was recorded as the trochlear angle and measured 15.4° and 16.0° in Cases 1 and 2, respectively. The anatomical lateral distal femoral angle (aLDFA) was measured according to previously described methods (9) and was 100.0° in Case 1 and 104.1° in Case 2. In Cases 1 and 2, additional screws placed to counteract medialising forces were removed on postoperative days 61 and 80, respectively; in Case 3, the fracture stabilization screws were removed at 193 days postoperatively due to implant prominence related to screw height and superficial positioning; no evidence of screw loosening or pull-out was identified. No cases required revision of the primary ridge fixation. In Case 3, the screws were removed with arthroscopic assistance, enabling direct visualization of the stifle joint with a minimally invasive approach. At the time of arthroscopy-assisted screw removal, increased joint effusion was noted radiographically. Arthroscopically, severe synovial hyperplasia and inflammation without significant cartilage and meniscal damage were noted, and the trochlear ridge showed no evidence of fragmentation or degeneration except a small step at the base of the medial trochlear ridge (Figure 3). The cranial cruciate ligament showed vascularization, with no signs of fraying, complete rupture, or mechanical instability. The screw head was embedded within abundant fibrous tissue, and no direct impingement between the screw head and the lateral joint capsule was observed. These findings suggested low-grade cranial cruciate ligament changes, likely secondary to chronic joint inflammation, without clinical signs of cranial drawer motion or functional cranial cruciate ligament insufficiency. In each case, serial radiographic and orthopedic evaluations were performed during the postoperative period. The final radiographic assessment was obtained on long-term follow-up: 488 days postoperatively in Case 1, 489 days in Case 2, and 377 days in Case 3 (corresponding to 184 days after implant removal). At the final follow-up, maintained positioning of the trochlear ridge fragment and stable implants were confirmed with no evidence of screw loosening or displacement in Cases 1 or 2. No sign of osteoarthritis was identified in any case at the final follow-up.

Subjective gait assessments showed clear improvement to grade 0 in all cases, with progressive return to normal weight-bearing gait within 82, 133, and 233 (corresponding to 40 days after implant removal) days postoperatively in Cases 1, 2, and 3, respectively. No further re-luxation of the patella was observed in any case during the follow-up period.

## 3 Discussion

Trochlear ridge fractures may occur as either intraoperative or postoperative complications associated with MPL surgery. Although this complication has been reported previously, descriptions of surgical stabilization strategies in small-breed dogs remain limited. The present report describes the management of trochlear ridge fractures identified intraoperatively or during revision surgery in three small-breed dogs and should be interpreted strictly as a technical description rather than evidence of efficacy or durability of the technique. Fixation configuration was not standardized and was adapted in each case according to fragment size, bone stock, and intraoperative stability.

In Cases 1 and 2, trochlear ridge fractures were not suspected preoperatively based on clinical examination and standard orthogonal radiography and were identified only at the time of revision surgery for recurrent MPL. Advanced imaging modalities, such as skyline radiography (10), ultrasonography (11), or computed tomography (12), have been reported to improve evaluation of trochlear morphology and may facilitate early detection of occult ridge injury. When recurrent MPL is suspected, particularly in cases with suspected trochlear involvement, the use of advanced imaging may improve preoperative planning and reduce intraoperative uncertainty.

In Case 3, an iatrogenic lateral trochlear ridge fracture occurred during primary surgery. This case highlights the technical demands of trochlear recession procedures, particularly in small-breed dogs, where limited bone volume and narrow anatomical corridors increase susceptibility to iatrogenic injury. Surgeons early in the learning curve may benefit from structured training using plastic bone models or cadaveric specimens to reduce procedural risks.

Block recession was performed in all cases for trochlear deepening. The depth and contour of trochlear recession may influence subchondral bone integrity, as excessive or asymmetric recession and sharp transitions at the base of the trochlear ridge may theoretically predispose the ridge to fracture, particularly in small dogs. Although block recession has been reported to maintain joint congruity and reduce postoperative complication rates (7), meticulous technique and preservation of subchondral support remain essential.

Lesions along the medial aspect of the trochlear groove may occur secondary to alignment discrepancies following trochlear recession procedures (13). In Cases 1 and 2, radiographic evaluation demonstrated deviation between the anatomical proximal femoral axis and the trochlear groove axis after trochleoplasty. As no validated reference values exist for this measurement, it was interpreted as a descriptive, exploratory indicator of femorotrochlear alignment rather than a causal factor. Although distal femoral varus deformity has been associated with patellar instability, its direct relationship to trochlear ridge fracture remains unproven. In these cases, the aLDFA met commonly cited radiographic thresholds for consideration of distal femoral osteotomy; however, the applicability of these criteria to small-breed dogs remains uncertain (9, 14), and corrective osteotomy was declined by the owners. The aLDFA was not quantified in Case 3, representing a limitation of this report, as the trochlear ridge fracture in that case was identified intraoperatively and considered iatrogenic. In more severe cases of trochlear damage, prosthetic trochlear groove or ridge replacement has been described as an alternative option to restore joint congruity and alignment (15, 16).

The fixation approach described was used to maintain fragment position in these cases and was associated with acceptable functional recovery in these cases during follow-up. This report is limited by the small sample size, heterogeneity in injury timing and fixation configuration, and the absence of objective outcome measures or biomechanical testing. Accordingly, the findings should be interpreted as descriptive observations. Further accumulation of cases and biomechanical evaluation is required to better define indications, fixation strategies, and long-term outcomes for trochlear ridge fractures associated with MPL surgery.

## Data Availability

The original contributions presented in the study are included in the article/supplementary material, further inquiries can be directed to the corresponding author.

## Footnotes

1 Mathemedix Oy, Espoo, Finland.

2 Suwa Ltd., Yamanashi, Japan.

3 Mizuho Co., Ltd., Chiba, Japan.

4 IMEX Veterinary Inc., Longview, TX, United States of America.

## References

[1] Arthurs GI Langley-Hobbs SJ. Complications associated with corrective surgery for patellar luxation in 109 dogs. Vet Surg. (2006) 35:559–66. doi: 10.1111/j.1532-959X.2006.00201.x, 16911156

[2] Slocum B Devine T. Partial carpal fusion in the dog. J Am Vet Med Assoc. (1982) 180:1204–8. 7085439

[3] Rossanese M German AJ Comerford E Pettitt R Tomlinson A de Vicente F. Complications following surgical correction of medial patellar luxation in small-to-medium-size dogs. Vet Comp Orthop Traumatol. (2019) 32:332–40. doi: 10.1055/s-0039-1692135, 30921826

[4] Cashmore RG Havlicek M Perkins NR James DR Fearnside SM Marchevsky AM . Major complications and risk factors associated with surgical correction of congenital medial patellar luxation in 124 dogs. Vet Comp Orthop Traumatol. (2014) 27:263–70. doi: 10.3415/VCOT-13-10-0151 24817090

[5] Chase D Farrell M. Fracture of the lateral trochlear ridge after surgical stabilisation of medial patellar luxation. Vet Comp Orthop Traumatol. (2010) 23:203–8. doi: 10.3415/VCOT-09-08-0105 20422125

[6] Putnam RW. Patellar luxation in the dog. Vet Clin North Am. (1968) 1:185–97.

[7] Johnson AL Probst CW Decamp CE Rosenstein DS Hauptman JG Weaver BT . Comparison of trochlear block recession and trochlear wedge recession for canine patellar luxation using a cadaver model. Vet Surg. (2001) 30:140–50. doi: 10.1053/jvet.2001.21852 11230768

[8] Huntingford JL Fossum T. "Fundamentals of physical rehabilitation" In: Fossum T, editor. Small animal surgery. 4th ed. Philadelphia: Elsevier (2013). 105–24.

[9] Swiderski JK Palmer RH. Long-term outcome of distal femoral osteotomy for treatment of combined distal femoral varus and medial patellar luxation: 12 cases (1999–2004). J Am Vet Med Assoc. (2007) 231:1070–5. doi: 10.2460/javma.231.7.1070, 17916032

[10] Garnoeva RS. Evaluation of trochlear dysplasia in dogs with medial patellar luxation-comparative studies. Acta Sci Vet. (2021) 49:1845. doi: 10.22456/1679-9216.115857

[11] Morishima T Tanegashima K Tomo Y Eto H Yamazaki A Edamura K. An assessment of trochlear groove morphology with the severity of medial patellar luxation using ultrasonography in dogs presented to a clinic in Japan. Vet Rec Open. (2025) 12:e70010. doi: 10.1002/vro2.70010, 40376249 PMC12078070

[12] Garnoeva RS. Computed tomography evaluation of morphological types of femoral trochlear dysplasia in small-breed dogs-a retrospective study. Vet Sci. (2025) 12:49. doi: 10.3390/vetsci12020049 39852924 PMC11768936

[13] van der Zee JH. Lesions in canine stifle joints due to trochleoplasties as treatment for medial patellar luxation. J S Afr Vet Assoc. (2015) 86:1245. doi: 10.4102/jsava.v86i1.1245, 26244588 PMC6138140

[14] Garnoeva RS Paskalev MD. Post-operative radiographic measures of pelvic limb alignment in dogs with medial patellar luxation after trochlear wedge recession versus trochlear block recession surgery. Vet World. (2021) 14:1504–10. doi: 10.14202/vetworld.2021.1504-1510 34316198 PMC8304435

[15] Dokic Z Lorinson D Weigel JP Vezzoni A. Patellar groove replacement in patellar luxation with severe femoro-patellar osteoarthritis. Vet Comp Orthop Traumatol. (2015) 28:124–30. doi: 10.3415/VCOT-14-11-0172, 25650724

[16] Nicetto T Longo F. Trochlear ridge prostheses for reshaping femoral trochlear ridges in dogs with patellar luxation. Vet Comp Orthop Traumatol. (2024) 37:98–106. doi: 10.1055/s-0043-1777717 37907244 PMC10932614

